# Rethinking Mistrust: Have We Misjudged the Complexity of African Americans' Relationship with Medical Research?

**DOI:** 10.1002/alz70860_105154

**Published:** 2025-12-23

**Authors:** Hector Salazar, Melita H Terry, Melissa L Knox, Susan M Sereika, Jennifer H Lingler

**Affiliations:** ^1^ University of Pittsburgh School of Nursing, Pittsburgh, PA, USA; ^2^ University of Pittsburgh Alzheimer's Disease Research Center (ADRC), Pittsburgh, PA, USA; ^3^ University of Pittsburgh, Pittsburgh, PA, USA

## Abstract

**Background:**

African Americans face significant disparities in Alzheimer's disease (AD) research participation, representing only 2% of clinical trial participants despite comprising 20% of the U.S. AD population (Manly and Glymour, 2021). These disparities stem from complex historical, social, and systemic barriers including medical mistrust, cultural insensitivity, and structural racism. We conducted a secondary analysis of data from the Recruitment Innovations for Diversity Enhancement (RIDE) study to investigate how sociodemographic variables (age, gender, income, and education) relate to trust in medical research among Black identifying individuals.

**Methods:**

RIDE was a multiphase research project that included a web‐based survey of 500 Black identifying participants from Southwestern Pennsylvania. Survey participants viewed materials from a culture‐centric storytelling campaign designed to enhance research engagement for Black individuals. Trust in medical researchers (possible range: 0‐84) was measured at baseline, prior to exposure to the narrative campaign materials.

**Result:**

Participants were 77.3% female, averaged 50.7 years of age, and had a mean 15.1 years of education. Participants’ mean rating of trust was 39.34 (range=15‐58; SD=6.95). Contrary to initial expectations that trust would vary among participants of different ages, income and educational levels or by gender, our comprehensive analyses using multiple linear regression and ANCOVA revealed no statistically significant findings. These analyses demonstrated no meaningful relationships between trust in medical researchers and sociodemographic variables of age, gender, income and education, including their interactions.

**Conclusion:**

This research highlights the complexity of understanding trust within African American communities, demonstrating that simple demographic categorizations are insufficient to explain research participation dynamics. The study underscores the need for more nuanced, culturally safe approaches to comprehending research engagement barriers.

